# Leakage of albumin in major abdominal surgery

**DOI:** 10.1186/s13054-016-1283-8

**Published:** 2016-04-26

**Authors:** Åke Norberg, Olav Rooyackers, Ralf Segersvärd, Jan Wernerman

**Affiliations:** Department of Anaesthesia and Intensive Care, Karolinska University Hospital Huddinge, Hälsovägen, SE-141 86 Stockholm Sweden; Department of Clinical Science, Intervention and Technology (CLINTEC), Karolinska Institutet, Hälsovägen, SE-141 86 Stockholm Sweden; Division of Surgery, Karolinska University Hospital Huddinge, Hälsovägen, SE-141 86 Stockholm Sweden

**Keywords:** Albumin, Inflammation, Transcapillary escape rate, Abdominal surgery

## Abstract

**Background:**

The time course of plasma albumin concentration (P-alb) and cumulative perioperative albumin shift as a measure of albumin extravasation in major abdominal surgery is not well described. Knowledge of these indices of the vascular barrier and vascular content are important for our understanding of fluid physiology during surgery and anesthesia.

**Methods:**

Patients (n = 10) were studied during esophageal or pancreatic surgery. P-alb was repeatedly measured over 72 h, and the mass balance of albumin and hemoglobin were obtained from measures of P-alb, blood hemoglobin and hematocrit.

**Results:**

P-alb decreased rapidly from baseline (32.8 ± 4.8 g/L) until the start of surgical reconstruction (18.7 ± 4.8 g/L; *p* < 0.001), and was thereafter stable until postoperative day 3. Cumulative perioperative albumin shift increased until 1 h after the end of surgery, when 24 ± 17 g (*p* < 0.001) had been lost from the circulation.

**Conclusions:**

The rapid fall in P-alb of more than 40 % consistently occurred during the first part of the surgical procedure, but albumin leakage progressed until 1 h after the end of surgery. After the initial drop, P-alb was stable for 72 h.

**Electronic supplementary material:**

The online version of this article (doi:10.1186/s13054-016-1283-8) contains supplementary material, which is available to authorized users.

## Background

The evidence behind existing guidelines for perioperative fluid therapy is surprisingly weak [1]. The recent reconsideration of the validity of the Starling’s principle of microvascular fluid shifts [2] calls for a new framework for our understanding of the vasculature. The perioperative time pattern of albumin plasma concentration and albumin mass balance will provide novel information on the vascular barrier and vascular content, two important aspects of the vasculature suggested in a recent review [3].

The decrease in plasma albumin concentration (P-alb) in association with surgical trauma is well known, and the mechanisms are probably multifactorial, but an increase in capillary leakage is thought to be a major component. This altered capillary leakage may be attributed to the inflammatory reaction elicited by the surgical trauma. This was suggested by Fleck et al. 30 years ago [4], when they were able to report an increase in postoperative transcapillary escape rate after cardiac surgery. In the same paper they described an even higher transcapillary escape rate in sepsis, another condition associated with an inflammatory reaction and hypoalbuminemia. Determination of albumin synthesis rate, both perioperatively as well as in critical illness, clearly indicates that the fractional synthesis rate is increased rather than decreased, in contrast to the lowering of plasma concentration [5]. Consequently, there must be a redistribution of albumin or a change in degradation rate.

In a previous study we reported a 35 % decrease in P-alb 2 days after major abdominal surgery compared to preoperative values [6]. However, we were unable to demonstrate any persistent elevation of albumin transcapillary escape rate on the second postoperative day, suggesting that capillary leakage had ceased at that time point.

The aim of the present study was to elucidate the time pattern of plasma albumin changes and calculated extravasation of albumin (the albumin shift) during and following standardized major abdominal surgery in an observational pilot study.

## Methods

The study was approved by the Regional Ethical Board in Stockholm (#2011/477), and was performed at the Karolinska University Hospital Huddinge between May and September 2011, in compliance with Good Clinical Practice and in accordance with the Declaration of Helsinki. The study was monitored by the Karolinska Trial Alliance. All patients gave written informed consent after being informed orally and in writing about the investigational procedure and the possible risks involved.

### Patients

Patients (n = 12) scheduled for major pancreatic (n = 9) or oesophageal surgery (n = 3) were recruited. One patient was excluded from further analysis because of an inoperable tumor and therefore a short surgical procedure, and one patient because exogenous albumin was given as colloid during surgery due to elevated plasma creatinine (presented as a case report, see Additional file 1: Text S1 and Additional file 2: Figure S1). The remaining 10 patients (2 females) were aged 65.7 ± 5.6 years and had a body mass index of 24.0 ± 2.3 kg/m^2^. The patients had a number of co-morbidities apart from their tumors: hypertension 4, diabetes mellitus 3, previous myocardial infarction 1, previous chemotherapy 2, congestive heart failure 2, atrial fibrillation 2, hypothyreosis 2, and one each of pancreatitis, trachea-esophageal fistula, multiple myeloma, multiple sclerosis, and deep venous thrombosis. The attending anesthesiologist graded preoperative health according to the American Society of Anesthesiologists’ (ASA) classification of physical health, all within grade II (n = 6) or III (n = 4). Two patients had a preoperative body weight loss of –6.8 and –10.0 kg over 1 and 2 months, respectively, corresponding to a weight loss of more than 10 % and a nutritional risk score of five [7]. The body weight of the remaining eight subjects differed by –0.6 kg (–1.8 to 0.5) from values 1–2 months preoperatively.

### Study procedure

P-alb, blood hematocrit (B-Htc), and blood hemoglobin (B-Hb) were measured 15 times over 3 days according to a predefined protocol. The first sample was taken as soon as an arterial line was established, then after induction of anesthesia, at the start of surgery, after 1 h of surgery, at the end of resection, at the start of reconstruction, at the end of surgery, 1, 2 and 3 h after surgery, on postoperative day 1 at 8 a.m., 10 a.m. and 4 p.m., on postoperative day 2 at 8 a.m., and on postoperative day 3 at 8 a.m.. Losses of albumin and hemoglobin due to bleeding, by suction and sponges, and postoperatively by drains were assessed by weighing and measurement of hemoglobin and albumin content. Losses of albumin in urine were considered as insignificant. Data from all intravenous infusions were collected. B-Hb was also measured in transfused donor blood and P-alb in donor plasma.

Anesthesia was performed according to the unit routines at the time of the study. Briefly, all patients had an arterial line, central venous catheter, thoracic epidural block with bupivacaine, fentanyl and epinephrine, and general anesthesia that after induction was maintained by sevoflurane in oxygenated air at an age-corrected minimal alveolar concentration of 0.8. Ventilation was adjusted to achieve normocapnia based on blood gas analyses. Intravenous fluids comprised starch (2 mL/kg/h; Volulyte®; Fresenius Kabi, Uppsala, Sweden), acetated Ringer’s solution (2 mL/kg/h), and glucose (25 mg/mL, 1 mL/kg/h) according to the unit’s routine. In this pragmatic study, bleeding was treated at the discretion of the attending anesthesiologist.

P-alb was analyzed by the Study Center at the Karolinska University Laboratory using nephelometry with a coefficient of variation of 1.9 % (IMMAGE® 800; Beckman Coulter AB, Bromma, Sweden), whereas B-Hb and B-Hct were assessed on a blood gas analyzer (ABL800 FLEX; Radiometer Medical ApS, Brønshøj, Denmark) at the Department of Anaesthesia.

### Calculations of mass balance

Baseline blood volume (BV) was calculated anthropometrically from gender and body size [8], and plasma volume (PV) was derived by:

1 $$ \mathrm{P}\mathrm{V}=\left(1{\textstyle\ \hbox{--}}\mathrm{B}{\textstyle \hbox{-}}\mathrm{H}\mathrm{c}\mathrm{t}\times 0.91\right)\times \mathrm{B}\mathrm{V} $$

The f-ratio 0.91 represents the ratio between total body hematocrit and large vessel B-Hct. The intravascular hemoglobin mass (MHb) is then:

2 $$ \mathrm{M}\mathrm{H}\mathrm{b} = \mathrm{B}\mathrm{V} \times \mathrm{B}\hbox{-} \mathrm{H}\mathrm{b} $$

By combining (1) and (2) we achieve:

3 $$ \mathrm{P}\mathrm{V}=\left(1{\textstyle \hbox{--}}\mathrm{B}{\textstyle \hbox{-}}\mathrm{H}\mathrm{c}\mathrm{t}\times 0.91\right)\times \mathrm{M}\mathrm{H}\mathrm{b}/\mathrm{B}{\textstyle \hbox{-}}\mathrm{H}\mathrm{b} $$

Intravascular albumin mass (IAM) can be similarly calculated by:

4 $$ \mathrm{I}\mathrm{A}\mathrm{M} = \mathrm{P}\mathrm{V} \times \mathrm{P}\hbox{-} \mathrm{alb} $$

Equations 1–4 are valid at all time points. When considering consecutive time points (*n* + 1 versus *n*) in the predefined protocol, MHb at time *n* + 1 can be calculated from the value at time *n* and measured loss and gain of hemoglobin in that time interval according to:

5 $$ {{\mathrm{MHb}}_{\mathrm{n}}}_{+1}={\mathrm{MHb}}_{\mathrm{n}}{\textstyle\ \hbox{--}}\mathrm{b}\mathrm{leeding}\mathrm{volume}\times \mathrm{mean}\mathrm{B}{\textstyle \hbox{-}}\mathrm{H}\mathrm{b}+\mathrm{transfusion}\mathrm{of}\mathrm{H}\mathrm{b} $$

When this MHb_*n*+1_ from equation (5) is inserted into equation (3) together with B-Hb_*n*+1_ and B-Hct_*n*+1_ it is possible to calculate PV_*n*+1_. This value can be inserted into equation (4) together with P-alb_*n*+1_ generating IAM_*n*+1_, representing albumin mass at time point *n* + 1 related only to B-Hb and B-Hct and measured loss and gain of hemoglobin. IAM’ represents another way to assess mass balance of albumin—directly by considering losses and gains of albumin over time. The apostrophe denotes that these IAM’ values are obtained differently. Gains are estimated from albumin content in plasma transfusions and albumin infusions, losses from measurement of albumin in drains or from estimated bleeding in suction bottles and sponges according to:

6 $$ \mathrm{Albumin}\mathrm{loss}=\mathrm{bleeding}\mathrm{volume}\times \mathrm{mean}\mathrm{P}{\textstyle \hbox{-}}\mathrm{alb}\times \left(1{\textstyle \hbox{--}}\mathrm{meanB}{\textstyle \hbox{-}}\mathrm{H}\mathrm{c}\mathrm{t}\right) $$

The cumulative difference between these two measures over time is presented in this paper as the cumulative perioperative albumin shift, supposedly to the extracellular space:

7 $$ \mathrm{Cumulative}\mathrm{perioperative}\mathrm{albumin}\mathrm{shift}=\mathrm{I}\mathrm{A}{\mathrm{M}}^{\prime }{\textstyle \hbox{--}}\mathrm{I}\mathrm{A}\mathrm{M} $$

Finally, the fractional plasma volume dilution at time *n* (fPVdil_*n*_) is then related to the baseline plasma volume PV_0_ according to:

8 $$ {\mathrm{fPVdil}}_n = {\mathrm{PV}}_n/{\mathrm{PV}}_0 $$

Similar fPVdil calculations serve as in-data in volume kinetic modelling [9].

### Statistics

Data are presented as mean ± standard deviation or median (range) as appropriate according to Sharpio Wilk’s *W* test of normality. Statistical evaluation was performed by repeated measures analysis of variance (ANOVA) followed by adjustment by Dunnett’s multiple comparison test when all time points were compared to baseline. When consecutive data time points were compared by ANOVA, Bonferroni correction was used to compensate for multiple comparisons. Friedman’s analysis of variance followed by Dunn’s correction for multiple testing versus baseline was used for nonparametric data. The software GraphPad Prism 5 was used for statistical calculations.

In a previous study of similar patients investigated under the same unit routines, the drop in P-alb from start of surgery to the second postoperative day was –11.5 ± 2.6 g/L [6]. This large and consistent drop, corresponding to an effect size of 4.4 (the difference divided by the standard deviation of the difference), made it likely that sufficient information of the time course would be obtained with a sample size of 10.

## Results

### Albumin mass balance

**Fig. 1:**
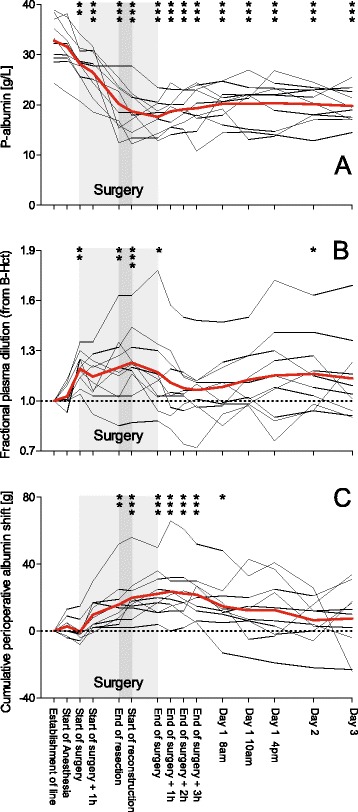
Temporal pattern of plasma albumin (*P-Albumin*) (**a**), fractional plasma dilution calculated from changes in blood hemoglobin and hematocrit (**b**), and cumulative perioperative albumin shift (**c**), in patients (n = 10) undergoing major abdominal surgery. *Bold line* represents the mean value. *Shaded area* represents time of surgery. Changes over time were assessed by analysis of variance (*p* < 0.0001 for **a** and **c**, and *p* = 0.0006 for **b**) followed by Dunnett’s multiple comparison test to compare all time points to baseline. *p < 0.05, **p < 0.01, ***p < 0.001. *B-Hct* blood hematocrit

P-alb decreased (*p* < 0.0001; Fig. 1a), mainly during the first part of the surgical procedure, from 32.8 ± 4.8 g/L at baseline to 18.7 ± 4.8 g/L (p < 0.001) at the start of reconstructive surgery, and to 19.8 ± 3.4 g/L (*p* < 0.001 compared with baseline) 3 days postoperatively (Fig. 1a) in subjects not treated with a continuous albumin infusion (n = 10). At the end of surgery, P-alb had decreased by 15.1 ± 5.2 g/L (95 % confidence interval of the mean decrease 11.4–18.8; *p* < 0.001) corresponding to 45 ± 12 % of the baseline value. Notably, there was a significant decrease in P-alb already at the start of surgery, as compared to baseline before the start of anesthesia.

Plasma volume increased (*p* = 0.0006; Fig. 1b) during surgery to reach its maximum at the start of the surgical reconstruction, but was postoperatively not different from baseline (Fig. 1b). Cumulative perioperative albumin shift changed (*p* < 0.0001; Fig. 1c) and the mean value increased throughout surgery to reach the most pronounced difference from baseline 1 h after the end of surgery (24 ± 17 g; 95 % confidence interval of the mean 11–36; *p* < 0.001), but from postoperative day 1 onwards did not differ from baseline (Fig. 1c). In total, six of the 10 patients were given 11 units of plasma during the study period containing a total of 88.2 g albumin. Four of those were given during the surgical procedure. Another 125 g albumin was given as albumin infusions, leaving only two subjects without any exogenous administration of albumin. Measured gains and losses of albumin are presented in Table 1. Similarly, in total, 15 units of erythrocyte concentrate with a mean hemoglobin of 192 g/L and a mean hematocrit of 0.59 were given to six different patients during the study period. The mass balance of albumin for four time periods is presented in Table 1, and cumulative albumin balance in Table 2. Laboratory tests are presented in Table 3.

**Table 1:** Albumin and fluid balance during four perioperative time periods in patients (n = 10) subjected to major abdominal surgery, and given starch as part of the perioperative fluid treatment

	Day of surgery, until EOS	EOS until POD 1 06:00	POD 1 until POD 2 06:00	POD 2 until POD 3 06:00
Albumin (g)				
Albumin loss^a^	–15.4 (–8.9; –45.9)	–4.1 (–0.9; –23.1)	–7.9 (–1.7; –16.9)	–4.7 (–0.9; -14.4)
Albumin infused	0 (0; 15.7)	0 (0; 17.0)	0 (0; 40)	0 (0; 40)
Albumin balance	–14.0 (–35.4; –1.6)	–3.3 (–11.8; 3.8)	–6.3 (–16.9; 30.7)	–3.0 (–11.1; 33.1)
Fluids (liters)				
Blood loss^a^	1.13 (0.48; 4.50)	0.30 (0.05; 1.59)	0.61 (0.13; 1.30)	0.46 (0.05; 1.31)
Urine	0.70 (0.13; 3.10)	0.87 (0.38; 1.66)	2.17 (1.15; 3.80)	2.54 (1.60; 4.92)
Sum of fluid losses	2.41 (0.61; 5.22)	1.45 (0.43; 2.32)	2.59 (1.53; 4.32)	3.05 (1.98; 5.48)
Albumin and blood products	0.13 (0; 1.53)	0 (0; 1.15)	0 (0; 0.49)	0 (0; 0.48)
Crystalloids + Glucose	2.60 (1.51; 3.76)	2.81 (1.01; 4.78)	2.80 (1.30; 4.37)	1.95 (1.00; 3.00)
Starch^b^	1.90 (0.60; 2.76)	0.63 (0; 1.03)	0.50 (0; 1.50)	0.50 (0; 1.00)
Sum of infusions	4.93 (2.11; 6.00)	3.87 (1.68; 5.55)	3.55 (1.50; 5.87)	2.37 (1.18; 4.50)
Fluid balance^c^	2.21 ± 0.81	2.22 ± 1.26	0.94 ± 1.55	–0.78 ± 1.47

Values are presented as median (range) or mean ± standard deviation, as appropriate

^a^Albumin and blood losses estimated from bleeding in suction bottles, sponges, and postoperatively in drains

^b^Macrodex is included in this volume used after vascular surgery in three patients amounting to 10 % of that volume

^c^Perspiration from wound, sweat, and breath is ignored

*EOS* end of surgery, *POD* postoperative day

**Table 2:** Cumulative balance of albumin, fluids and body weight from baseline before surgery until third postoperative day in patients (n = 10) subjected to major abdominal surgery

Cumulative balance	End of surgery	POD 1 06:00	POD 2 06:00	POD 3 06:00	*p* ^a^
Albumin (g)	–17.7 ± 11.8**	–20.7 ± 9.1^b^	–20.9 ± 17.5^c^	–20.3 ± 21.8^b^	0.0004
Fluids (liters)	2.21 ± 0.81**	4.43 ± 1.09^c^ **	5.38 ± 1.97^c^	4.59 ± 2.33^c^	<0.0001
Body weight (kg)		2.9 ± 1.4^c^ ***	3.8 ± 1.7^c^	3.2 ± 2.7^c^	<0.0001

Values are presented as mean ± standard deviation. Starting from the preoperative baseline (zero), data were analyzed simultaneously using repeated measures ANOVA with Bonferroni correction for simultaneously multiple testing of differences between consecutive time points and differences from baseline

^a^ *p* values refer to the ANOVA of all data

**p < 0.01, ***p < 0.001, versus previous time point; ^b^ *p* < 0.01 compared to baseline; ^c^ *p* < 0.001 compared to baseline

*POD* postoperative day

**Table 3:** Laboratory tests in patients (n = 10) subjected to major abdominal surgery

	Baseline	POD 1 06:00	POD 2 06:00	POD 3 06:00	*p*
Blood hemoglobin (g/L)	125 ± 16	106 ± 10***	102 ± 12***	105 ± 14 ***	<0.0001^a^
Blood hematocrit (fraction)	0.384 ± 0.048	0.328 ± 0.032***	0.316 ± 0.037***	0.323 ± 0.043 ***	<0.0001^a^
Plasma C-reactive protein (mg/L)	1 (<1; 76)	63 (9; 125)*	114 (6; 246)***	116 (10; 270) ***	<0.0001^b^
P-creatinine (μmol/L)	62 ± 11	67 ± 17	61 ± 12	60 ± 14	0.202^a^

Values are presented as mean ± standard deviation or median (range)

^a^Repeated measures analysis of variance with Dunnett’s correction for multiple testing versus baseline

^b^Friedman’s repeated measures analysis of variance with Dunn’s correction for multiple testing versus baseline

*p < 0.05, ***p < 0.001, compared to baseline

*POD* postoperative day

### Surgery and fluid balance

Surgery time was 369 ± 84 min. Further details of fluid balance during and after the surgical procedures are given in Tables 1 and 2. Five patients had pancreatoduodenectomy, one of these also had splenectomy and vascular reconstruction; one patient had distal pancreatic resection with hemicolectomy and splenectomy, one patient had pancreatic resection with resection of the upper mesenteric artery, one patient had reconstruction of the oesophagus, one patient had transthoracic resection of the oesophagus, and one patient had transhiatal resection of the oesophagus. Three patients (oesophageal resection in two and pancreatic resection in one) started with enteral feeding on postoperative day 1, whereas the other patients remained fasting, only drinking small amounts of water.

Gains and losses of fluid and the resulting fluid balance over four time periods during 3 perioperative days are also presented in Table 1. Cumulative fluid balance changed over time (*p* < 0.0001; Table 2) and, when consecutive time points were analyzed simultaneously, both preoperative versus end of surgery, and end of surgery compared with postoperative day 1 differed significantly (*p* < 0.01), even after Bonferroni correction as presented in Table 2. On postoperative day 2 body weight gain reached its highest value of 3.8 ± 1.7 kg (*p* < 0.001; Table 2), and cumulative fluid balance was then 5.4 ± 2.0 L (*p* < 0.001; Table 2). At the same time point the correlation between fluid balance and weight gain did not reach statistical significance (*p* = 0.091, *r*^2^ = 0.316), neither did cumulative perioperative albumin shift correlate with body weight gain (*p* = 0.336, *r*^2^ = 0.12) or fluid balance (*p* = 0.206, *r*^2^ = 0.19).

## Discussion

In this observational pilot study we demonstrate that the major part of the perioperative decrease in P-alb consistently occurred during the first part of the surgical procedure before the start of the surgical reconstruction, i.e., during the initial 253 ± 50 min of surgery. After that time P-alb was stable throughout the study period of 3 days.

Although albumin extravasation might promote edema and weight gain, there is no evidence as yet that this is actually the case, nor that it is important for patient outcome. In contrast, a low P-alb on the first postoperative day has been demonstrated to be an important risk factor after major abdominal surgery [10, 11]. Many factors might contribute to postoperative weight gain, such as the degree of inflammation, differences in the surgical trauma, high- or low-risk anastomoses to the pancreas, effects of epidural block, and so forth. In our pragmatic pilot study protocol, infusions of albumin and crystalloids were given at the discretion of the attending doctors, rather than following a strict study protocol criteria. Therefore, it is not surprising that a correlation between cumulative albumin shift and fluid balance or weight gain could not be demonstrated in our group of patients.

### Mechanisms of hypoalbuminemia

The mechanisms of the P-alb decrease during surgery are unclear; they are most likely multifactorial, and might even be variable over time. The rapid fall in P-alb before surgery might be related to general sympathetic tone, posture, vasodilation caused by the epidural block, or effects on blood pressure attributed to general anesthesia. There is an extravasation of albumin and other plasma proteins in anesthetized or septic animals of different species after infusion of a variety of different fluids such as whole blood [12], albumin [13], and crystalloids [14]. Three major reasons behind this extravasation are discussed: overhydration, increased capillary leakage, and change in albumin turnover.

#### Overhydration

When large fluid volumes are infused over a short period of time in healthy subjects that are unlikely to have any substantial volume deficit, there is a pronounced extravasation [15]. It is speculated that a rapid intravenous infusion causes overhydration and thereby a “washout” of the endothelial surface layer causing an increased leakage of albumin and other plasma constituents [16]. In our patients, the routine use of starch substitution (2 mL/kg/h) might have contributed to the expansion of the plasma volume (Fig. 1b), and indirectly to albumin extravasation. The increase in body weight and a positive fluid balance indicates that our patients had some degree of overhydration.

#### Inflammation

Plasma leakage is strongly associated with inflammation [17], but the time of onset is not well established in conjunction with surgery. In a pivotal paper on albumin leakage, the transcapillary escape rate of albumin measured with radio-iodine is doubled 3–6 h after thoracic surgery compared to preoperative values [4]. This rise precedes the increase in laboratory markers of inflammation, such as C-reactive protein, alpha-1 acid glycoprotein, and fibrinogen evoked by surgery [18]. In contrast, interleukin-6 reaches its peak in plasma at the end of major abdominal surgery and decreases significantly over the following 6 h [19]. Infusion of endotoxin is insufficient to induce increased vascular permeability in volunteers [20], whereas sepsis is associated with a greater and more persistent elevation of biomarkers than after major surgery [19]. The inflammatory response was only assessed by C-reactive protein in our patients. The role of inflammation compared to that of overhydration in explaining albumin leakage after major surgery warrants further research.

#### Change in albumin turnover

Changes in turnover, i.e., synthesis or catabolism of albumin, are sparsely investigated in connection with surgery, but the absolute synthesis rate is only marginally decreased [6, 21, 22]. However, considering that the turnover rate is about 5 % per day, changes in turnover cannot possibly have any major impact on the quick albumin shifts seen over just a few hours.

### Mass balance of albumin and exploration of confounders

The changes in intravascular albumin mass were calculated from changes in hemoglobin concentration, and demonstrated to be in accordance with plasma protein loss after major gynecological surgery [23]. However, the method used has been criticized for insufficient precision in determination of albumin balance which will propagate to the calculation of cumulative perioperative albumin shift. To determine the possible impact of this level of precision, simulations were performed over possible confounders. Anthropometric blood volume calculations, uncertainty in the f-ratio between total body hematocrit and B-Hct, variation of this ratio over time, the difficulty in accurately assessing the losses of hemoglobin in the clinical setting of major abdominal surgery, and the impact of uncertainty in P-alb and B-Hb measures were evaluated. These five possible sources of error influence the magnitude of the changes of the albumin shift versus time plot, but not the direction or time pattern (see Additional file 3: Text S2; Additional file 4: Figure S2; Additional file 5: Figure S3; Additional file 6: Figure S4).

Apart from small analytical errors of P-alb, short-term effects on P-alb (minutes) are likely associated with simultaneous changes in plasma volume evoked by changes in body position [24], or states of under- or overhydration. Exogenous vasopressors such as norepinephrine are associated with plasma volume contraction [25], and we speculate that physiological stress can also have similar rapid effects. Intermediate effects on P-alb (hours) include compromised lymphatic return of albumin, either by para-aortal resection of lymph glands in major abdominal surgery or by altered lymphatic outflow pressure (i.e., central venous pressure) caused by positive pressure ventilation [26], the state of hydration, or posture [27]. Also, the transcapillary escape rate can be increased by overhydration or inflammation [4]. Long-term changes in P-alb (days to months) can be associated with increased synthesis (steroids), decreased synthesis (malnutrition or liver failure) [28], increased losses (inflammatory bowel disease or renal failure) [28], or increased volume of distribution (obesity or ascites). However, these long-term factors are less likely to affect the drop in P-alb over a couple of hours described in this paper.

The strength of this pilot study lies in the presentation of new knowledge on the time course of albumin leakage during major abdominal surgery while keeping track of gains and losses. The robustness of the results in a small group of subjects is demonstrated by simulation over confounders. Among limitations of the study are the limited external validity related to the utilized fluid routine with starch (now abandoned), that blood substitution was left to the discretion of the attending anesthesiologist, and the difficulty in assessing bleeding correctly in such a complicated situation as open abdominal surgery. Still, the temporal aspects of capillary leakage, as reflected by the albumin concentration and the albumin shift, are important and need to be considered when perioperative fluid regimens are studied. Here, our study adds original information.

## Conclusions

P-alb decreases by 43 % during the initial part of major abdominal surgery and is thereafter stable. This corresponds to a cumulative perioperative albumin shift of 24 g (95 % confidence interval 11–36 g) 1 h after the end of surgery. This consistent early perioperative redistribution of albumin is hypothesized to be attributed mainly to overhydration and a change in capillary leakage, and the underlying mechanisms and the contribution of each factor warrants being addressed in future studies.

## Key messages

- P-alb decreased by 43 % during the initial part of major abdominal surgery and was thereafter stable up to 72 h.
- Cumulative perioperative albumin shift, i.e., losses not accounted for by bleeding or other changes in plasma albumin mass, reached 24 ± 17 g 1 h after the end of surgery.

## Additional files

Additional file 1: Text S1. A case report of albumin infusion compared to starch. (DOCX 13 kb) Additional file 2: Figure S1. Temporal pattern of P-albumin, plasma dilution, and cumulative perioperative albumin shift. (EPS 66 kb) Additional file 3: Text S2. Simulations over five possible confounders in the albumin mass balance calculations. Text, references and figure legends. (DOCX 25 kb) Additional file 4: Figure S2. Simulations over the impact of different assumptions on cumulative perioperative albumin shift. (EPS 73 kb) Additional file 5: Figure S3. Simulations of imprecise assessments of bleeding. (EPS 73 kb) Additional file 6:Figure S4. Simulations of measurement imprecision of P-alb and B-Hb. (EPS 59 kb)

## Abbreviations

ANOVA: analysis of variance
B-Hb: blood hemoglobin
B-Hct: blood hematocrit
BV: blood volume
fPVdil: fractional plasma volume dilution
IAM: intravascular albumin mass (determined from changes in B-Hb and B-Hct)
IAM’: intravascular albumin mass (determined from losses and gains of albumin)
MHb: intravascular hemoglobin mass
P-alb: plasma albumin concentration
PV: plasma volume
