# Supplementary material for: Leakage of albumin in major abdominal surgery
Source: Crit Care. 2016 Apr 26;20:113. doi: 10.1186/s13054-016-1283-8 (PMC4845320; doi:10.1186/s13054-016-1283-8)
Supplement: Additional file 1: Text S1. — A case report of albumin infusion compared to starch. (DOCX 13 kb) [file 13054_2016_1283_MOESM1_ESM.docx]

# **Additional file 1. Text S1**

# **A case report of albumin infusion during abdominal surgery compared to starch**

In the main paper one subject was excluded from analysis because albumin solution was given as routine volume infusion during surgery instead of starch, which constitutes another situation. This was caused by an elevated preoperative plasma creatinine, 128 µmol/L. In this supplementary text, data from that patient is presented and compared to the mean of the 10 patients in the main publication.

In the other 10 patients intravenous fluids comprised starch (2 mL/kg/h; Volulyte®, Fresenius Kabi, Uppsala, Sweden), acetated Ringer’s solution (2 mL/kg/h), and glucose (25 mg/mL, 1 mL/kg/h) according to the unit’s routine at the time of the study. The patient in this report received Albumin 50 mg/mL (2 mL/kg/h) instead of starch. Otherwise, the anesthetic procedures were identical to that of the other patients.

The time pattern of the single patient that received albumin instead of starch differed in several aspects, as presented in the Figures S1A-C. A higher P-Alb was maintained on the day of surgery, compared to the mean of the starch patients, but eventually fell to the same low values postoperatively that applied to the other patients (Figure S1A). This subject received infusion of Albumin 50 mg/ml 1000 ml during the surgical procedure together with 2 units of erythrocytes, and another 28 g of albumin as plasma transfusion and albumin infusion post-operative day 2 together with one more erythrocyte unit. A more marked calculated dilution of plasma was seen compared to starch patients (Figure S1B), and the albumin shift was negative during most of surgery, as opposed to all other subjects, but became positive after an albumin infusion post-operative day 2 (Figure S1C).

This latter finding seems physiologically unlikely, but a cross check of data gave no further explanation to this result. As our simulations in Additional file Text S2 suggest, there are several possible confounders, although data still seem to keep a reasonable robustness.

In conclusion, the patient supplemented with albumin instead of starch showed some differences from the other patients, but as a single case must be interpreted with great caution.

# **Figure Legend**

**Figure S1.** Temporal pattern of P-Albumin (A), fractional plasma dilution calculated from changes in blood hemoglobin and hematocrit (B), and cumulative peri-operative albumin shift (C), in one patient receiving albumin, dotted black line, compared to the mean value of 10 patients receiving starch during major abdominal surgery, bold red line. Shaded area represents time of surgery.
