# Supplementary material for: Leakage of albumin in major abdominal surgery
Source: Crit Care. 2016 Apr 26;20:113. doi: 10.1186/s13054-016-1283-8 (PMC4845320; doi:10.1186/s13054-016-1283-8)
Supplement: Additional file 3: Text S2. — Simulations over five possible confounders in the albumin mass balance calculations. Text, references and figure legends. (DOCX 25 kb) [file 13054_2016_1283_MOESM3_ESM.docx]

# **Additional file 3. Text S2**

# **Simulations over five possible confounders in the albumin mass balance calculations**

1) The anthropometric blood volume calculations in healthy volunteers has a standard error of 0.4 L [1], and others state the coefficient of variation to be 11-12% [2]. Simulations show some impact on magnitude but not on time pattern of albumin shift (Figure S2A).

2) The uncertainty in the f-ratio between total body hematocrit and B-Hct has been reported as 0.91 ± 0.03 over a wide range of B-Hct values [3], and the International Committee for Standardization in Hematology endorses a value of 0.90 [4]. In a recent series of consecutive patients submitted for blood volume measurements a median F-ratio of 0.92 (0.82-1.09) was found [5]. A few studies address F-ratio in isovolemic hemodilution during anesthesia [6, 7]. Simulations over different ratios show little impact on the cumulative perioperative albumin shift plots (Figure S2B).

3) The assumption that the chosen ratio between central and total body B-Hct is constant over time is sparsely addressed in literature. During anesthesia an increase from 0.829 ± 0.050 to 0.925 ± 0.073 has been reported at 30 min after preoperative volume loading with 20 mL/kg albumin 5% at a rate of 90 mL/min in 20 gynecological patients, compared to baseline [7]. Hemodialysis is also associated with an equilibration of total body Hct and large vessel B-Htc [8]. The time course of this ratio during anesthesia and surgery in our patients is unknown, but an attempt to investigate the issue by simulation of 15 curves with randomly assigned ratios normally distributed around 0.91 (random numbers achieved in excel N(0.91;0.04)) is presented in Figure S2C.

4) To accurately quantify losses of B-Hb through bleeding is difficult in the clinical setting of major abdominal surgery. Clotting in suction bottles, fluids splashed over the hands of surgeons to facilitate knotting, bile, rinsing of the wound, perspiration from bowels or liver surface, and many more confounders contribute to errors. Also postoperative bleeding that does not present in drains is hard to capture. However, simulations of misinterpreted bleeding shows that the impact on cumulative perioperative albumin shift is almost negligible (Figure S3A) whereas fractional dilution of plasma volume is grossly affected (Figure S3B).

5) A fifth source of variability comes from the measures of P-alb, B-Hb, and B-Hct is this dynamic situation. P-alb by the nephelometry method has an error of 0.5 g/L and B-Hb an error of 2 g/L. B-Hct was assumed to correlate to B-Hb. By random generation of normality distributed errors of such magnitude we simulated 10 new subjects and the resulting impact on the cumulative perioperative albumin loss is presented in Figure S4. The standard deviation of the albumin loss was approximately 4 g at all time points. Pre-analytical errors might be even larger, but we have no reason to expect such mistakes. In summary these five possible errors influence the magnitude of the changes of the albumin shift versus time plot, but not the direction or time pattern.

# **References**

1. Nadler SB, Hidalgo JH, Bloch T. Prediction of blood volume in normal human adults. Surgery. 1962;51:224-32.

2. Hurley PJ. Red cell and plasma volumes in normal adults. J Nucl Med. 1975;16:46-52.

3. Chaplin H, Jr., Mollison PL, Vetter H. The body/venous hematocrit ratio: its constancy over a wide hematocrit range. J Clin Invest. 1953;32:1309-16.

4. Recommended methods for measurement of red-cell and plasma volume: International Committee for Standardization in Haematology. J Nucl Med. 1980;21:793-800.

5. Moralidis E, Papanastassiou E, Arsos G, Chilidis I, Gerasimou G, Gotzamani-Psarrakou A. A single measurement with (51)Cr-tagged red cells or (125)I-labeled human serum albumin in the prediction of fractional and whole blood volumes: an assessment of the limitations. Physiol Meas. 2009;30:559-71.

6. Haller M, Brechtelsbauer H, Akbulut C, Fett W, Briegel J, Finsterer U. Isovolemic hemodilution alters the ratio of whole-body to large-vessel hematocrit (F-cell ratio). A prospective, randomized study comparing the volume effects of hydroxyethyl starch 200,000/0.62 and albumin. Infusionsther Transfusionsmed. 1995;22:74-80.

7. Rehm M, Haller M, Orth V, Kreimeier U, Jacob M, Dressel H, et al. Changes in blood volume and hematocrit during acute preoperative volume loading with 5% albumin or 6% hetastarch solutions in patients before radical hysterectomy. Anesthesiology. 2001;95:849-56.

8. Dasselaar JJ, Lub-de Hooge MN, Pruim J, Nijnuis H, Wiersum A, de Jong PE, et al. Relative blood volume changes underestimate total blood volume changes during hemodialysis. Clin J Am Soc Nephrol. 2007;2:669-74.

# **Figure Legends**

**Figure S2.** Simulations over the impact of different assumptions on cumulative peri-operative albumin shift in grams. Panel A shows different start estimates for anthropometric blood volume, 0.8 – 1.25 * volume according to Nadler [1]. Bold line is 0.8. Panel B shows different values for the f-ratio between total body and large vessel hematocrit 0.83 – 0.87 – 0.91 – 0.95 and 1.0. Bold line is 1.0. Panel C shows 15 cases with randomized values of f-ratio N(0.91 ; 0.04) in each time point. Bold line is median.

**Figure S3.** Simulations of imprecise assessments of bleeding on cumulative peri-operative albumin shift (panel A) and fractional plasma volume dilution (panel B) calculated from B-Hb and B-Hct. Bold line represents a subject with pancreatduodenectomy that had an estimated peri-operative bleeding of 1.5 L, thin dotted line represents 50% of that bleeding and thin continuous line 200%. Bold dotted line represents the effects on albumin shift and plasma dilution of a 0.5 L daily postoperative undetected bleeding.

**Figure S4.** Simulations of measurement imprecision of P-alb (sd. 0.5 g/L) and B-Hb (sd. 2 g/L) on cumulative perioperative albumin shift, calculated form B-Hb and B-Hct and compared to actual albumin loss by mass balance. Bold line represents mean, dotted bold line the curve around which the simulations were performed, thin dotted line represents 10 simulated subjects.
